# The Medical Council and Midwives

**Published:** 1898-04-16

**Authors:** 


### The Medical Council and Midwives.

A special session of the General Medical Council was convened for the 5th instant, at the request of the Privy Council, in order to report to the Government on the provisions of Mr. J. B. Balfour's Bill for the Registration of Midwives before that measure came up for a second reading in the House of Commons. The first business of the Council was to elect a president in the room of the late Sir Richard Quain, with the result that Sir William Turner was unanimously chosen. His long experience of the work of the Council, coupled with his sagacity and urbanity, render the choice an admirable one; even apart from the consideration that it is essentially gratifying to the Scottish members. Sir William then proposed and carried that Sir Dyce Duckworth and Mr. Brudenell Carter should be associated with him as a Committee for the preparation of a suitable resolution of condolence with the family of the late President; and, this done, the special business of the meeting was proceeded with.

The Council had previously appointed a Committee to report upon the provisions of any Bill relating to midwives which might be referred to it by the Government, and this Committee presented a report which, however, could only be described as a very unsatisfactory and inconclusive document. Some members of the Committee were of opinion that the terms of the reference to them only required them to prepare a *précis* of the Bill, setting forth the nature of its provisions, and the way in which it proposed to accomplish the objects of its promoters; while other members thought themselves called upon to advise the Council as to the nature and scope of recommendations which should be made to the Government upon the question. Before this point was settled, and while two drafts of a report were under discussion, the Chairman, Sir Richard Thorne-Thorne, was unfortunately disabled by illness from further attendance, and the most experienced obstetrician among the members, Dr. Lombe Atthill, was also unable to come to the two last meetings. In the Committee thus reduced there were two dissentients from the report, which was received, but not adopted, by the Council.

In the Council itself, the discussion was opened by a somewhat lengthy speech from Dr. McVail, who proposed a resolution condemnatory of the Bill in its entirety, and who was seconded by Sir Christopher Nixon. It was, perhaps, a little odd that the lead in opposition, to a Bill not intended to apply to either Scotland or Ireland, should thus be taken by a Scotch and an Irish member; and, after some discussion, the resolution was by permission deferred until a later period of the proceedings, and the report of the Committee was approached, with the intention of taking it clause by clause. The hour for adjournment was reached before even the first clause had been fully dealt with; but, in the meanwhile, the views of many members of the Council had been incidentally stated with reference to all the questions involved, and Professor Sir William Gairdner had thrown out the suggestion that perhaps midwives might be "licensed" rather than "registered." It was felt by the majority of the Council that this suggestion would furnish a practical solution of many difficulties. It was feared by many that "registration," as urged by some opponents of the Bill, would, in reality, establish an inferior and dangerous order of medical practitioners, and would thus be in direct contravention of the Medical Act of 1886; and it was also thought that, while it might be exceedingly difficult to obtain from Parliament any definite prohibition, under penalties, of the practice of "unregistered" midwives, there would be less difficulty, and no violation of precedent, in prohibiting the practice of "unlicensed" ones; such a prohibition being already in force with regard to other trades, the designations of which are not protected. Anyone who pleases may call himself a cabdriver; but no one who has not a license is permitted to drive a cab for hire. Acting on what appeared to be the general feeling of the Council, the Chairman of the Business Committee, Professor MacAlister, prepared a series of recommendations prior to the meeting of the second day, and these, after discussion, were finally accepted by the Council. They are to the effect that no person, not qualified and registered under the Medical Acts, should be permitted to practise midwifery for gain except under the sanction of an annual license; that a Board should be appointed to determine, subject to the approval of the Medical Council, the conditions under which licenses should be granted, and the offences for which they should be revoked; that the regulations so framed and approved should be issued to local sanitary authorities by the department of State charged with the supervision of the public health, and that arrangements should be made by which these local sanitary authorities were rendered administrators of the chief provisions of the suggested law. In so far as Mr. Balfour's Bill was inconsistent with these recommendations, the Council expressed disapproval of it, and a hope that it might not be accepted by Parliament. There can be little doubt that, even without the opposition of the Council, it would be defeated on the second reading on account of the indefinite charges which it would cast on county and borough rates; but it may fairly be hoped that the Government, in some future and not distant session, may see its way to introduce and carry a measure on the lines which the Council has laid down, and by which this long-standing controversy will be finally terminated.

